# Evaluation of a biohybrid photoelectrochemical cell employing the purple bacterial reaction centre as a biosensor for herbicides

**DOI:** 10.1016/j.bios.2014.02.050

**Published:** 2014-08-15

**Authors:** David J.K. Swainsbury, Vincent M. Friebe, Raoul N. Frese, Michael R. Jones

**Affiliations:** a School of Biochemistry, Medical Sciences Building, University of Bristol, University Walk, Bristol BS8 1TD, United Kingdom; b Division of Physics and Astronomy, Department of Biophysics, VU University Amsterdam, De Boelelaan 1081, Amsterdam 1081 HV, The Netherlands

**Keywords:** Biosensor, Herbicide, Reaction centre, Photocurrent, Photovoltaics, Atrazine

## Abstract

The *Rhodobacter sphaeroides* reaction centre is a relatively robust and tractable membrane protein that has potential for exploitation in technological applications, including biohybrid devices for photovoltaics and biosensing. This report assessed the usefulness of the photocurrent generated by this reaction centre adhered to a small working electrode as the basis for a biosensor for classes of herbicides used extensively for the control of weeds in major agricultural crops. Photocurrent generation was inhibited in a concentration-dependent manner by the triazides atrazine and terbutryn, but not by nitrile or phenylurea herbicides. Measurements of the effects of these herbicides on the kinetics of charge recombination in photo-oxidised reaction centres in solution showed the same selectivity of response. Titrations of reaction centre photocurrents yielded half maximal inhibitory concentrations of 208 nM and 2.1 µM for terbutryn and atrazine, respectively, with limits of detection estimated at around 8 nM and 50 nM, respectively. Photocurrent attenuation provided a direct measure of herbicide concentration, with no need for model-dependent kinetic analysis of the signal used for detection or the use of prohibitively complex instrumentation, and prospects for the use of protein engineering to develop the sensitivity and selectivity of herbicide binding by the *Rba. sphaeroides* reaction centre are discussed.

## Highlights

• The *Rhodobacter sphaeroides* reaction centre was used as a biosensor for herbicides.
• Herbicide concentration was assessed through the attenuation of a photocurrent.
• The biosensor showed selectivity for triazine herbicides.
• The limit of detection of the biosensor was in the low nanomolar range.
• Photocurrent attenuation is a simple and direct basis for a herbicide biosensor.

## 1 Introduction

There is increasing concern over the effects of pesticides on human health and the well-being of the biosphere, creating a need to fully understand the consequences of exposure of non-target species to these chemicals (Köhler and Triebskorn, 2013). This concern has also fuelled a desire for convenient and relatively low-cost devices for in-field and real-time monitoring of contamination of non-target sites by specific chemical agents. One solution is to develop portable biosensors that are specific to a particular pesticide molecule, or family of molecules, by combining a biological component that interacts with the molecule(s) with a high affinity and selectivity, and a supporting apparatus that can accurately transduce the consequences of that interaction for quantification.

Much of modern agriculture is supported by the use of herbicides to control competing growth of weed species. A variety of chemicals show selective herbicidal activity, with many inhibiting the light-powered electron transfer used by plants to generate the NADPH and ATP that support carbon fixation, cellular growth and division, the mode of action being competitive inhibition of quinone reduction and protonation by the Photosystem II (PSII) reaction centre (Draber et al., 1991). The use of tens of thousands of tonnes of such agents annually across the globe is not without controversy, a case in point being the triazine herbicide atrazine (*N*^2^-ethyl-6-chloro*-N*^4^-isopropyl-1,3,5-triazine-2,4-diamine) that is used to inhibit the growth of annual and perennial monocotyledonous and dicotyledonous weeds through both pre- and post-emergence treatment. The use of atrazine in European Union (EU) countries was discontinued in 2003 amid concerns over its persistence and accumulation in soil and watercourses (see EU report SANCO/10496/2003 (2014) – URL given in reference list). However it is still used extensively in the United States, particularly for the production of corn, sorghum and sugar cane, and in around sixty other countries worldwide. Despite having not been licensed for use in the EU for 10 years this molecule and its metabolites are persistent in water and soil, and it remains a major contaminant of environmental samples (Jablonowski et al., 2011). Some related triazine herbicides such as simazine are also not permitted for use in the EU but others, such as terbuthylazine (*N*^2^-tert-butyl-6-chloro-*N*^4^-ethyl-1,3,5-triazine-2,4-diamine), are still licensed and are used extensively.

The case of atrazine is one of the better known controversies regarding the use of herbicides to improve crop production and quality. There is growing evidence in support of claims that atrazine can disrupt the endocrine systems of a wide range of species including amphibians, fish, reptiles and mammals, leading to a range of gonadal abnormalities including hermaphrodism, feminisation of males, reproductive organ malformation and reduction in sperm quality (Victor-Costa et al., 2010; Hayes et al., 2011; Jablonowski et al., 2011; Köhler and Triebskorn, 2013). In particular, atrazine has been associated with gonadal abnormalities in male frogs (Hayes et al., 2002, 2010, 2011). Controversies of this sort have provided much of the impetus for the development of biosensors that are specific for herbicides such as atrazine, and there have been multiple attempts at using PSII-containing biomaterials in a biosensor aimed at this molecule, and herbicides from the same family. A variety of preparations have been employed, ranging from intact cells and organelles to the purified PSII reaction centre; see for reviews (Giardi et al., 2001; Giardi and Pace, 2005; Tibuzzi et al., 2007; Campàs et al., 2008; Scognamiglio et al., 2010). Inhibition of photosynthetic electron transfer through PSII generates a range of detectable signals, including decreases in oxygen consumption, decreases in electron flow and increases in fluorescence (Giardi et al., 2001; Giardi and Pace, 2005; Tibuzzi et al., 2007). The more sensitive prototype biosensors have yielded values of half maximal inhibitory concentration (IC_50_) and limits of detection (LOD) in the low nanomolar range, similar to the upper concentration limit for an individual pesticide in drinking water in the EU of 0.1 µg L^−1^, equating to ~2 nM for a molecule the size of atrazine. In addition to addressing many challenges concerning the portability, sensitivity and stability of PSII-based prototype biosensors, in recent years attention has turned towards improving their specificity for particular herbicides through modelling and protein engineering (Rea et al., 2009; Scognamiglio et al., 2010; Lambreva et al., 2013), and the design of peptides that mimic quinone binding sites (Scognamiglio et al., 2013). Herbicides that act through mechanisms other than inhibition of electron flow through PSII are not detected by this type of biohybrid system.

**Fig. 1:**
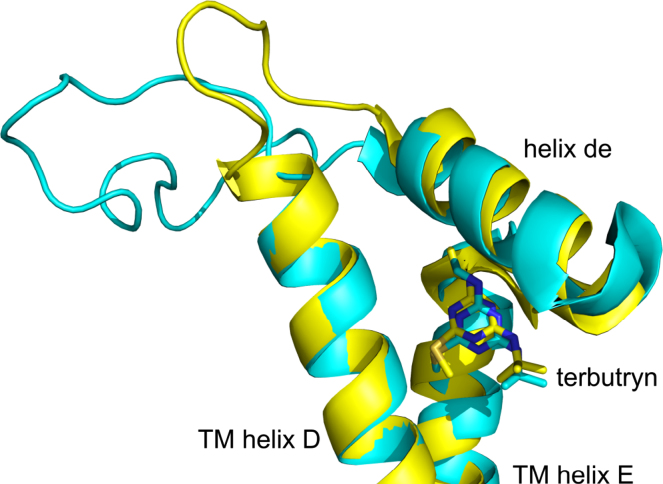
Overlay of the D, E and de ɑ-helices that form the Q_B_ pocket, and terbutryn occupying the pocket in the *Rba. sphaeroides* reaction centre (yellow (online) or light-grey (print)) and the *T. elongatus* PSII (cyan (online) or dark-grey (print)). Prepared using Protein Data Bank entries 2BNP (Katona et al., 2005) and 3PRQ (Broser et al., 2010), and PyMOL (Schrödinger, LLC). For a stereo, colour representation see Supplementary Fig. 1.

An alternative to PSII for the development of herbicide-specific biosensors is the homologous reaction centre from purple photosynthetic bacteria, electron transfer through which is also blocked by triazines and some other classes of herbicide (Stein et al., 1984). The quinone reductase (Q_B_) site in both complexes is formed by the transmembrane (TM) D and E ɑ-helices, and the connecting de ɑ-helix (Fig. 1), and the binding of the very potent triazine herbicide terbutryn has been characterised in both Photosystem II (Broser et al., 2010) and the purple bacterium *Rhodobacter* (*Rba*.) *sphaeroides* (Katona et al., 2005). The overlay in Fig. 1 shows the strong similarity in backbone fold around the Q_B_ site in the two complexes and consistency in the binding conformation adopted by terbutryn (and see Supplementary Fig. 1 for a colour, stereo view of this overlay). Purple bacterial reaction centres from species such as *Rba. sphaeroides* offer a number of advantages as an experimental system for the development of biosensors, not the least of which is the fact that they are expressed at high levels during chemoautotrophic growth in the dark, facilitating extensive protein engineering without compromising the viability of the organism (Jones, 2009). The *Rba. sphaeroides* reaction centre is also relatively robust, one reason being that it operates at potentials that are much less oxidising than PSII, and thus it is far less prone to self-inflicted photo-oxidative damage.

Compared to PSII there have been fewer evaluations of the use of purple bacterial reaction centres to detect herbicides. Most studies have utilised the fact that charge separation between the primary electron donor bacteriochlorophyll pair (P) and the primary (Q_A_) and secondary (Q_B_) acceptor quinones is blocked at the stage P^+^Q_A_^-^ in the presence of a herbicide, displacement of the Q_B_ quinone preventing formation of P^+^Q_B_^-^. Recombination of the P^+^Q_A_^-^ radical pair occurs around 10-fold more rapidly than recombination of P^+^Q_B_^-^ (half times of ~100 ms and ~1 s, respectively) and so the binding of a herbicide to the Q_B_ site will alter the kinetics of radical pair recombination after a period of illumination, or the kinetics of P photo-oxidation during a period of weak illumination, both of which can be monitored through recovery or bleaching, respectively, of a P ground state absorbance band. Several research groups have used this to study binding of herbicides or other inhibitors by purified *Rba. sphaeroides* reaction centres in solution (Jockers et al., 1993; Spyridaki et al., 2000; Baldini et al., 2003; Andreu et al., 2005), reconstituted into liposomes (Peters et al., 1997), or embedded in a cationic polymer (Mallardi et al., 2007; Giustini et al., 2012). Data analysis in most of these studies required the application of a mathematical model to translate changes in the kinetics of P photooxidation or subsequent recovery into the amount of herbicide binding at the Q_B_ site (e.g. see Andreu et al. (2005) and Giustini et al. (2012)). Atrazine binding has also been detected by surface plasmon resonance (SPR) through an unidentified change in the properties of *Rba. sphaeroides* reaction centres immobilised on an SPR chip (Nakamura et al., 2003).

In previous work we described a simple photoelectrochemical cell based on purified *Rba. sphaeroides* reaction centres interfaced with an unfunctionalised gold electrode, and characterised the capacity of such cells to generate photocurrents under a range of conditions of illumination, applied potential and mediator concentration (den Hollander et al., 2011). Photocurrents of several hundred nA cm^−2^ could be generated using reaction centres interfaced with the working and counter electrode by cytochrome *c* and ubiquinone, respectively. In the present work we explore the use of such a photoelectrochemical cell as a biosensor by quantifying photocurrent attenuation by a range of herbicides and other quinone site inhibitors.

## 2 Materials and methods

### 2.1 Construction and expression of His-tagged reaction centres

DNA encoding a ten residue polyhistidine tag preceded by a thrombin cleavage site was designed in silico such that the resulting protein sequence was LALVPRGSSAHHHHHHHHHH. The DNA sequence was placed before the *pufM* stop codon in plasmid pUCXB-1, which is a derivative of pUC19 containing a 1841 bp *Xba*I–*Bam*HI fragment encompassing *pufLM* (McAuley-Hecht et al., 1998), such that the protein sequence was inserted at the C-terminus of the PufM polypeptide. The resulting *Xba*I–*Bam*HI fragment was shuttled into plasmid pRKEH10D (Jones et al., 1992a), which is a derivative of broad-host-range vector pRK415 containing a 6.2 kb *Eco*RI–*Hin*dIII fragment encoding *pufQLM*. The resulting plasmid was named pvLMt, and was introduced into *Rba. sphaeroides* strain DD13 (Jones et al., 1992b) by conjugative transfer, as described previously (Jones et al., 1992a). Transconjugant strains were selected as described previously (Jones et al., 1992a,b) and contained His-tagged reaction centres as the sole pigment–protein complex in the cell.

### 2.2 Purification of His-tagged reaction centres

The procedure for purification of His-tagged reaction centres was based on that described by Goldsmith et al. (1996). *Rba. sphaeroides* DD13 expressing plasmid pvLMtX was grown under dark/semiaerobic conditions in M22 medium as described previously (Jones et al., 1994). Cells from 12 L of culture medium were harvested by centrifugation and resuspended in 100 mL 20 mM Tris (pH 8), containing several crystals of DNAse I and two cOmplete Protease Inhibitor Cocktail Tablets (Roche). Cells were lysed using a Constant Systems cell disruptor at 20,000 psi and cell debris was removed by centrifugation at 26,890*g* for 15 min at 4 °C. Reaction centres were isolated from membranes in the supernatant by the addition of 0.5% n-dodecyl-N,N-dimethylamine-N-oxide (LDAO) and 200 mM NaCl in a final volume of 500 ml 20 mM Tris (pH 8), followed by stirred incubation in the dark at 4 °C for 1 h. Membrane debris was removed by centrifugation at 100,000*g* for 30 min at 4 °C. Solubilised reaction centres in the supernatant were loaded onto a 20 ml HisPrep FF Ni-NTA column (GE Healthcare) pre-equilibrated with 100 mL of 20 mM Tris (pH 8), containing 300 mM NaCl, 0.1% LDAO and 10 mM imidazole (equilibration buffer). The column was washed with 400 mL equilibration buffer to remove unbound protein and reaction centres were eluted with 20 mM Tris (pH 8.0)/300 mM NaCl/0.1% LDAO/500 mM imidazole. Eluted reaction centres were further purified by size exclusion chromatography on a Superdex 200 16/60 column (GE Healthcare) pre-equilibrated with 20 mM Tris (pH 8)/0.1% LDAO. Purity was assessed by UV/vis absorbance spectroscopy (Okamura et al., 1974), fractions with a ratio of protein absorbance at 280 nm to bacteriochlorophyll absorbance at 802 nm of less than 1.4 being retained for use. Reaction centre concentrations were calculated using an extinction coefficient at 802 nm of 2.88×10^5^ M^−1^ cm^−1^ (Straley et al., 1973).

### 2.3 Fabrication of photovoltaic cells

Photovoltaic cells were constructed as described by den Hollander and co-workers (den Hollander et al., 2011). A 2 mm diameter gold working electrode was cleaned by polishing with a slurry of 0.3 µm aluminium oxide nanopowder followed by sonication for 10 min in Milli-Q water (Millipore). In addition, electrochemical cleaning was performed by immersion in 1 M sulphuric acid and cycling of applied potential between −0.3 and +1.5 V vs a Radiometer Analytical standard calomel electrode (SCE) using an Autolab PGSTAT128N potentiostat (Metrohm) controlled by a PC running Autolab Nova 1.4 software (Metrohm). The electrode was then rinsed with Milli-Q water and dried under a stream of nitrogen gas.

To adhere reaction centres the cleaned gold electrode was immersed in 25 µL of a 1 mg mL^−1^ (approximately 10 µM) solution of purified reaction centres in 20 mM Tris (pH 8.0)/0.1% LDAO for 1 h at 4 °C in dark. Unbound reaction centres were removed by dipping the electrode in ice-cold 20 mM Tris (pH 8.0) three times. The coated gold electrode was assembled into a glass-bottomed cell along with a Pt counter electrode (Radiometer Analytical), and an Ag/AgCl reference electrode (Radiometer Analytical). The cell was filled with 1 ml of 20 mM Tris (pH 8.0) containing 20 µM horse heart cyt *c* and 30 µM UQ_0_ (both from Sigma-Aldrich) and placed above a shutter-controlled 1.5 W 880 nm LED light source (Roithner LaserTechnik), resulting in an illumination intensity of 46 mW cm^−2^ at the working electrode. A potential of −100 mV vs SCE was applied and photocurrents measured by chronoamperometry using the Autolab PGSTAT128N potentiostat (Metrohm) controlled by the PC running Autolab Nova 1.4 software (Metrohm).

### 2.4 Determination of the *K*_*m*_^(apparent)^ for UQ_0_

Photovoltaic cells were assembled as described above but with the omission of UQ_0_. Photocurrents of 30 s duration were measured in the absence of UQ_0_ and after the addition of increasing amounts of UQ_0_. Maximal currents from three titrations were averaged, plotted as a function of UQ_0_ concentration then fitted with the Michaelis–Menten equation using Origin 8.0 (OriginLab).

### 2.5 Inhibition of photocurrents and inhibitor titrations

For single measurements at high concentrations of herbicide or inhibitor, the photocurrent from a freshly prepared cell was measured for a standard period of 30 s, as described above, and then 2 µL of 50 mM atrazine, terbutryn or stigmatellin, or 10 µl of 50 mM bentazon, bromoxynil, DCMU or capsaicin dissolved in ethanol was added. The cell was incubated for 1 min before measurement of a 30 s photocurrent. All herbicides and inhibitors were purchased from Sigma-Aldrich, and their structures are shown in Supplementary Fig. 2.

For titrations, the photocurrent from a freshly prepared cell was measured for 30 s before and after the addition of increasing concentrations of atrazine, terbutryn or stigmatellin prepared in 20 mM Tris (pH 8.0)/20 µM cyt *c*/30 µM UQ_0_. After each addition the cell was equilibrated for 60 s before measuring the photocurrent, which was sufficient to allow stabilisation of the applied current required to maintain a potential of −100 mV vs SCE. Maximal photocurrents were plotted as a function of inhibitor concentration and fitted with a logistic function to determine the IC_50_ and IC_5_ (see text). These IC_50_ values were used to estimate *K*_*i*_ using the Cheng–Prusoff equation (see text).

### 2.6 Analysis of recombination kinetics

Analysis of the effects of herbicides or inhibitors on the kinetics of charge recombination was performed using a Cary60 spectrophotometer connected to an external 1 cm cuvette holder (Ocean Optics) via a fibre-optic coupler. A white light pulse of 50 ms duration was applied to the sample cuvette at 90° to the pulsed measuring beam by an HL-2000-FHSA white light source with a fast shutter (Ocean Optics) delivering approximately 25 W m^−2^ illumination at the cuvette surface. The actinic pulse was triggered by a TGP110 pulse generator (Thurlby Thandur Instruments). Solutions of 13.2 µM reaction centres in 20 mM Tris (pH 8.0)/ 30 µM UQ_0_ were prepared with and without the addition of 500 µM atrazine, terbutryn, stigmatellin, bromoxynil, bentazon, capsaicin or DCMU. Test samples were loaded into to a 3×3 mm fluorescence cuvette (Hellma) aligned to the measuring and excitation pulses and absorbance at 865 nm was measured for 20 s after delivery of the light pulse, with a further period of at least 60 s to allow full dark adaptation of the reaction centres before re-excitation. A set of eight kinetic traces were recorded for each sample, averaged, and fitted using a single or double exponential function in Origin 8 (OriginLab).

## 3 Results

### 3.1 Fabrication of photoelectrochemical cells

Photoelectrochemical cells were fabricated using wild type *Rba. sphaeroides* reaction centres that were modified with a poly-histidine tag on the C-terminus of the M-polypeptide, and purified by nickel affinity chromatography (see Section 2). Purified reaction centres were adhered to 2 mm diameter cleaned gold electrodes and inserted into a detergent-free buffer solution containing 20 μM cyt *c* and 30 µM UQ_0_ as mediators (see Section 2). As reported previously, these electrodes generated cathodic photocurrents of the order of −1 to −2 µA cm^−2^ under the measuring conditions described in Section 2 (den Hollander et al., 2011). The operational lifetime of an individual coated electrode was well in excess of that required to conduct a full herbicide titration (data not shown).

To optimise the concentration of the substrate for the reaction centre quinone reductase (Q_B_) site in the cell the *K*_*m*_^(apparent)^ for UQ_0_ was determined by measuring the maximum photocurrent density during a 30 s period of illumination as a function of increasing concentrations of UQ_0_. A concentration-dependent increase in the photocurrent amplitude was obtained, which was fitted with the Michaelis–Menten equation (Supplementary Fig. 3). The *K*_*m*_^(apparent)^ in the photoelectrochemical cell was 42±2 µM and the maximum current density was −1.1 µA cm^−2^. This *K*_*m*_^(apparent)^ for UQ_0_ is, to our knowledge, the strongest reported to date. Previously published *K*_*D*_ values appear to be dependent on the LDAO concentration (Gerencsér and Maróti, 2008), being 600 µM in 0.1% LDAO, 480 µM in 0.06% LDAO and 175 or 90 µM in 0.0025% LDAO (McComb et al., 1990; Paddock et al., 1988; Gerencsér and Maróti, 2008). As the present assay was conducted in detergent-free buffer, the stronger *K*_*m*_^(apparent)^ obtained is consistent with this trend.

The sensitivity of such photoelectrochemical cells to inhibitors was dependent on two key variables, the maximal photocurrent and the concentration of substrate. As both of these parameters were directly dependent on the concentration of UQ_0_, assays of herbicide inhibition of photocurrent were conducted in the presence of 30 µM UQ_0_. This gave high sensitivity to inhibition whilst retaining a maximal uninhibited photocurrent density of up to −1 µA cm^−2^.

### 3.2 Inhibition of photocurrent generation

The response of photocurrent generation to different classes of herbicide was initially tested using a relatively high concentration. Agents screened were the triazide herbicides atrazine and terbutryn, the thiadiazine bentazon, the nitrile bromoxynil, the phenylurea 3-(3,4-dichlorophenyl)-1,1-dimethylurea (DCMU – also known as diuron) and the non-herbicides capsaicin and stigmatellin (structures shown in Supplementary Fig. 2). Stigmatellin, an antibiotic isolated from the myxobacterium *Stigmatella aurantaica* (Kunze et al., 1984), is a potent inhibitor of the Q_B_ site in the *Rba. sphaeroides* reaction centre (Bibikov et al., 1994) with a dissociation constant in the 4–400 nM range depending on conditions (Gerencsér et al., 2004). An X-ray crystal structure for *Blc. viridis* reaction centres with this inhibitor bound is available (Lancaster and Michel, 1997) and its inhibitory effects are well characterised. Capsaicin, the component that gives chilli peppers their distinctive properties, has been reported to be a weak inhibitor of the *Rba. sphaeroides* reaction centre and again an X-ray crystal structure with this agent in the Q_B_ pocket has been determined (Spyridaki et al., 2000).

**Fig. 2:**
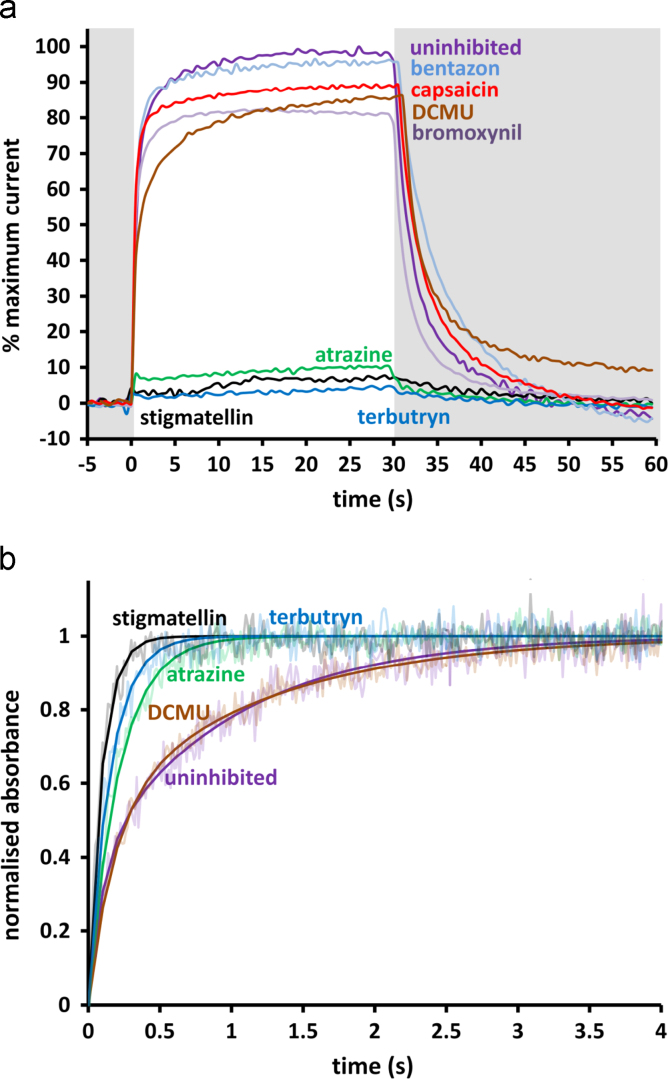
Effects of relatively high concentrations of herbicides on reaction centre function. (a) Impact on photocurrent density. To adjust for small variations in absolute current output from cell to cell, traces are normalised to the maximum photocurrent generated by each cell over an identical time period before the addition of the herbicide or inhibitor. Grey background indicates periods without illumination. (b) Effect on charge recombination in photo-excited reaction centres, measured through recovery of primary donor absorbance at 865 nm after photobleaching with a pulse of white light. Raw data (faded lines) are overlaid with fitted single or double exponential decays (solid lines). Data for bentazon, bromoxynil and capsaicin, which were similar to those for the uninhibited and DCMU samples, have been omitted for clarity. Parameters from fits are shown in Supplementary Table 1.

The results of an initial screen using a high concentration of each agent are summarised in Fig. 2a. Almost complete abolition of the photocurrent (less than 10% remaining) was achieved with 100 µM atrazine, terbutryn and stigmatellin. In contrast very little inhibition (greater than 80% remaining) was obtained with 500 µM bentazon, bromoxynil, capsaicin and DCMU.

To ensure this pattern of results was not specific to the conditions within the photoelectrochemical cell, the ability of these inhibitors to replace ubiquinone at the Q_B_ site was also tested by measuring the rate of recombination from Q_A_ and Q_B_ to the primary donor bacteriochlorophyll (P) after excitation with a pulse of white light (see Section 2). As outlined in Section 1, recombination of P^+^Q_A_^−^ or P^+^Q_B_^−^ occurs with lifetimes of ~100 ms and ~1 s, respectively. As the Q_B_ quinone is partially lost during purification of reaction centres, assays were conducted in the presence of 30 µM UQ_0_ to reconstitute Q_B_, the reaction centre concentration being 13.2 µM. The recombination rate was unaffected by the presence of 500 µM DCMU (Fig. 2b) or the same concentrations of bentazon, bromoxynil and capsaicin (not shown), but recombination was accelerated significantly in the presence of 500 µM atrazine, terbutryn or stigmatellin (Fig. 2b), suggesting near to complete Q_B_ inhibition. These assays therefore correlated with those monitoring photocurrents, strong inhibition being seen only for stigmatellin, atrazine and terbutryn. Parameters from fits to the decay data in Fig. 2b (and equivalent measurements for bentazon, bromoxynil, DCMU and capsaicin, not shown) are compiled in Supplementary Table 1.

### 3.3 Determination of IC_50_ and *K*_*i*_ for atrazine, terbutryn and stigmatellin

**Fig. 3:**
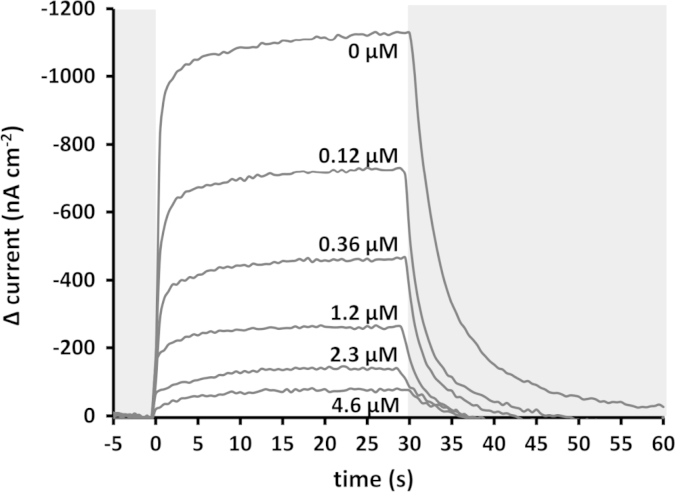
Photocurrent attenuation by increasing concentrations of terbutryn. Grey background indicates periods without illumination.

**Fig. 4:**
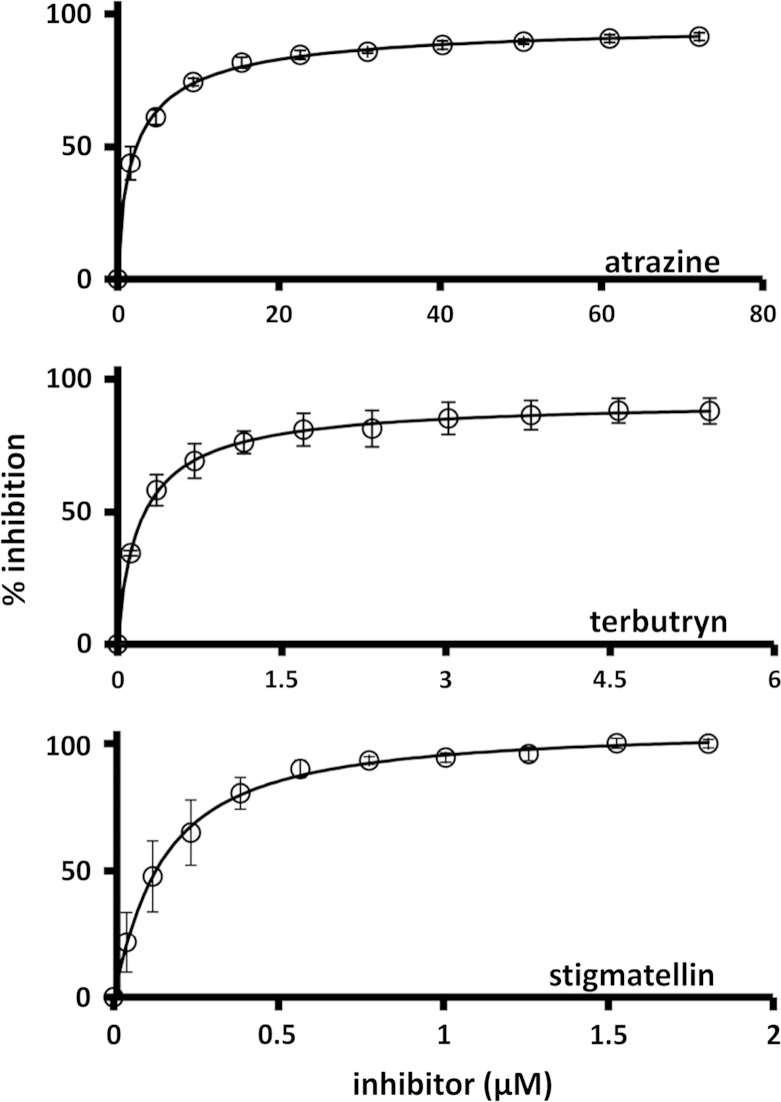
Inhibition of photocurrent generation by atrazine, terbutryn and stigmatellin. Percentage inhibition is the maximum photocurrent density during 30 s illumination relative to that obtained prior to addition of the first aliquot of inhibitor. Each data point is an average from three titrations on three separate cells, with error bars showing the standard error. Solid lines show fits to a logistic function to determine the IC_50_ (see text).

To quantify the sensitivity of photocurrent generation to atrazine, terbutryn and stigmatellin the maximal photocurrent after a standard period of 30 s was recorded as a function of herbicide or inhibitor concentration. A sample titration for terbutryn is shown in Fig. 3 and titration data from three repeats are shown in Fig. 4. The titration data, expressed as the percentage of the maximum current in the absence of the herbicide/inhibitor, were fitted to a logistic function (Eq. (1)) to determine the IC_50_.

(1) $$Y=\;\mathrm{Min}\;+\frac{\mathrm{Max}\;-\;\mathrm{Min}}{1+{\left(\left[\mathrm{Inhibitor}\right]{/\text{IC}}_{50}\right)}^{-\mathrm{HillCoefficient}}}$$

where Max and Min are maximal and minimal photocurrents, respectively. The values of IC_50_ obtained were similar for sigmatellin and terbutryn at 280±60 nM and 208±10 nM, respectively. The IC_50_ for atrazine was approximately 10-fold higher at 2.1±0.1 µM (Table 1). Given the level of noise in the raw data we estimate the sensitivity with which a change in current can be detected is in the region of 5% of the amplitude of the uninhibited current. Based on this, the LOD can be represented by an IC_5_ value that can be calculated from the parameters of the IC_50_ fit (Eq. (2)). This yielded values of 49, 8.3 and 10 nM for atrazine, stigmatellin and terbutryn, respectively.

(2) $${\mathrm{IC}}_{5}=\left({\left(\frac{5}{100-5}\right)}^{1/\text{HillCoefficient}}\right){\mathrm{IC}}_{50}$$

To compare the sensitivity of our photochemical cell device to previous studies employing *Rba. sphaeroides* reaction centres, the IC_50_ values were converted to values for the binding affinity of the inhibitor (*K*_*i*_) using the Cheng–Prusoff equation (Eq. (3)) (Cheng and Prusoff, 1973)

(3) $$K_{i}=\frac{{\mathrm{IC}}_{50}}{1+([S]/K_{m})}$$

where [*S*] is the substrate concentration and *K*_*m*_ is the measured Michaelis constant for UQ_0_ (see above). The values obtained for atrazine, terbutryn and stigmatellin were 1.2 µM, 123 nM and 165 nM, respectively (Table 1).

## 4 Discussion

### 4.1 Photoelectrochemical cells with *Rba. sphaeroides* reaction centres can form the basis of a triazine-specific biosensor

In an early study, Stein and co-workers conducted a comprehensive screen of the efficacy of PSII herbicides from different classes in inhibiting the Q_B_ site in the *Rba. sphaeroides* reaction centre, and concluded that both benzonitriles and symmetrical triazines are effective inhibitors (Stein et al., 1984). Effectiveness was quantified as a dissociation constant for herbicide binding at the Q_B_ site, using an experimental system comprising 1 μM reaction centres in a buffer containing 10 mM Tris (pH 8.0), 100 mM NaCl, 0.06% TX-100 and 20 μM UQ_10_. *K*_*D*_ values for effective triazine inhibitors were in the range 3 μM (for terbutryn) to 120 μM (for atrazine). Bromoxynil, a benzonitrile, yielded a *K*_*D*_ of 30 µM and was classed as an effective inhibitor whereas DCMU, an urea, was classed as an ineffective inhibitor with a *K*_*D*_ in excess of 800 µM.

In the present study the two symmetrical triazine herbicides tested were found to be potent inhibitors of the Q_B_ site whereas DCMU was ineffective, in line with the conclusions of Stein et al. (1984). However in the present experimental system bromoxynil was ineffective, in contrast to the data of Stein et al. where its efficacy was intermediate between that of terbutryn and atrazine. The reasons for this are not clear, but may be related to differences in experimental conditions such as the use of 0.06% Triton X-100 in the buffer system in the previous work compared to the detergent-free buffer used in the present study. In support of this, it has been shown that the affinity of quinone for the Q_B_ site is sensitive to detergent concentration (McComb et al., 1990). We also note that Sinning and co-workers grouped the related ioxynil with DCMU as very weak inhibitors of the *Blc. viridis* reaction centre (Sinning et al., 1989). In the present study only weak inhibition was seen with bentazon, a thiadiazine herbicide that, to our knowledge, has not previously been tested on purple bacterial reaction centres. Our conclusion, therefore, is that photoelectrochemical cells constructed from *Rba. sphaeroides* reaction centres can form the basis of a triazine-specific biosensor.

An advantage of using a reaction centre-generated photocurrent as a sensor for herbicides is that the measurement is direct, with no requirement for mathematical manipulation of the measured signal or assumptions over parameters such as the rate of recombination of different charge separated states. Detection of herbicides through attenuation of currents supported by PSII has been carried out in a small number of studies, with most looking at atrazine and so enabling comparison with the present report. Bhalla and co-workers looked at the effect of atrazine on currents generated by PSII reaction centres or BBY particles (PSII-enriched membranes) adhered to screen printed gold electrodes, reporting an IC_50_ of 49 nm and LOD of 1.15 nM (Bhalla and Zazubovich, 2011). Koblížek and co-workers looked at atrazine inhibition of currents obtained from PSII complexes in a glutaraldehyde–BSA matrix on a graphite electrode, reporting an IC_50_ of 90 nm and LOD of 2 nM (Koblížek et al., 2002). Masojídek and co-workers used PSII adhered to a screen printed Pt working electrode in a glutaraldehyde–BSA–glycerol matrix, reporting an IC_50_ of 890 nM (Masojídek et al., 2011). Finally, Touloupakis and co-workers looked at spinach thylakoids immobilised on a graphite working electrode in an albumin glutaraldehyde matrix, reporting an IC_50_ of 100 nM and LOD of 12.7 nM (Touloupakis et al., 2005). Thus the values of IC_50_ and LOD obtained with PSII, ranging from 49 to 890 nM and 1 to 2 nM, respectively, are considerably lower than the 2.1 µM IC_50_ and 49 nM LOD obtained in the present study using *Rba. sphaeroides* reaction centres. Some studies of herbicide detection using PSII have reported a limit of detection for atrazine of a few nanomolar, which is in the region of the maximum permitted amount of an individual pesticide in the EU.

### 4.2 Scope for tuning of the herbicide selectivity or sensitivity of a biosensor based on the *Rba. sphaeroides* reaction centre

A drawback of using PSII as a biosensor is that it exhibits strong binding across a range of different herbicide types and variants, and so is not very selective. This compromises its usefulness as a biosensor for specific herbicide such as atrazine. To address this, site-directed or random mutagenesis of the region around the Q_B_ binding pocket in PSII has been carried out with a view to increasing selectivity for individual triazine herbicides (Rea et al., 2009; Lambreva et al., 2013). An advantage possessed by the *Rba. sphaeroides* reaction centre in this regard is that it appears to be natively selective for this class of herbicide, and as illustrated below there seems to be every prospect that this selectivity could be enhanced or switched to a different class of herbicide through protein engineering.

A second issue is the sensitivity with which herbicides can be detected by the purple bacterial RC. The values of LOD estimated in the present study for atrazine (49 nM) and terbutryn (8.3 nM) are somewhat lower than those previously reported in studies of herbicide binding by *Rba. sphaeroides* reaction centres (750–3000 nM and 40–170 nM, respectively) (Jockers et al., 1993; Andreu et al., 2005; Giustini et al., 2012), but are nevertheless considerably higher than the maximum concentration for a single herbicide permitted in drinking water in the EU (~2 nM for atrazine). As a result if a biosensor based on purple bacterial reaction centres is to be useful in detecting triazine herbicides at around this threshold level then its sensitivity needs to be improved by around an order of magnitude for terbutryn and two orders of magnitude for atrazine. Again, there seems every prospect that this could be brought about by protein engineering of the Q_B_ pocket to be more akin to that seen in PSII, where the affinity for these herbicides is significantly greater (see above). This is entirely feasible in the case of *Rba. sphaeroides* because a fully functioning reaction centre is not required to support its growth, and so any mutations made in this part of the protein are not constrained by a need to retain a functional ubiquinone reductase site.

A further advantage is that engineering of the purple bacterial reaction centre for enhanced and/or selective herbicide binding can be guided by X-ray crystal structures that show how herbicides are bound into the Q_B_ pocket. Such structures are available in the Protein Data Bank for the *Rba. sphaeroides* reaction centre with terbutryn (Katona et al., 2005), the *Blc. viridis* reaction centre with terbutryn (Lancaster et al., 2000), atrazine (Lancaster and Michel, 1999) or stigmatellin (Lancaster and Michel, 1997), and PSII with terbutryn (Broser et al., 2010). As an illustration of how site-directed mutagenesis can be used to address selectivity and/or sensitivity of herbicide binding by the reaction centre, Sinning and co-workers have shown that the affinity of *Blc. viridis* reaction centres for the normally weakly-binding DCMU can be increased by at least four orders of magnitude through a single tyrosine to phenylalanine change in a residue close to the Q_B_ binding pocket (Sinning et al., 1989). This *Blc. viridis* mutant was isolated following exposure of cells to terbutryn, the most potent of the triazine inhibitors of bacterial reaction centres, sensitivity to this inhibitor decreasing by 660-fold (with a similar decrease for atrazine). This rather conservative mutation also increased sensitivity to ioxynil (a benzonitrile similar to bromoxynil) by 100-fold. This study showed rather dramatically the considerable scope for tailoring the inhibitor sensitivity and selectivity of the Q_B_ site in the purple bacterial reaction centre through site-directed mutagenesis. We are currently exploring the key determinants for binding of herbicides such as terbutryn and atrazine through molecular modelling.

## 5 Conclusions

The data described in this report illustrate the direct manner in which the photocurrent generate by *Rba. sphaeroides* reaction centres adhered to an unfunctionalised gold electrode could provide the basis of a biosensor for controversial herbicides such as atrazine and its relatives. The construction of such a device will require further development of the hardware for photoexcitation and current detection, and protein engineering of the biological component to provide greater selectivity to individual herbicide molecules and sensitivity to low concentrations of such agents. Given the tractability and relative robustness of the purple bacterial reaction centre there seems every prospect that the sensitivity and selectivity of this component can be engineered to lie in the range required for implementation of a practical device.

## Figures and Tables

**Table 1:** Sensitivity of photocurrent generation to effective Q_B_ inhibitors.

**Inhibitor**	**IC**_**50**_^a^ (nM)	**Calculated*****K***_**i**_^b^ (nM)	**LOD**^c^ (nM)
Atrazine	2100±100	1200	49
Terbutryn	208±10	123	8.3
Stigmatellin	280±60	165	10

a Values are given ±standard error.

b Based on *K*_*m*_ for UQ_0_ of 43 µM and a UQ_0_ concentration of 30 µM using the Cheng–Prusoff equation (see text).

c Calculated LOD based on the IC_50_ and Hill coefficient derived from the fit parameters (see text).

## References

[bib1] Andreu Y., Baldini F., Giannetti A., Mencaglia A. (2005). Talanta.

[bib2] Baldini F., Domenici C., Giannetti A., Masci D., Mencaglia A. (2003). Sens. Actuators B.

[bib3] Bhalla V., Zazubovich V. (2011). Anal. Chim. Acta.

[bib4] Bibikov S.I., Bloch D.A., Cherepanov D.A., Oesterhelt D., Semenov A.Yu (1994). FEBS Lett..

[bib5] Broser M., Gabdulkhakov A., Kern J., Guskov A., Muh F., Saenger W., Zouni A. (2010). J. Biol. Chem..

[bib6] Campàs M., Carpentier R., Rouillon R. (2008). Biotechnol. Adv..

[bib7] Cheng Y., Prusoff W.H. (1973). Biochem. Pharmacol..

[bib8] den Hollander M.-J., Magis J.G., Fuchsenberger P., Aartsma T.J., Jones M.R., Frese R.N. (2011). Langmuir.

[bib9] Draber W., Tietjen K., Kluth J.F., Trebst A. (1991). Angew. Chem. Int. Ed..

[bib10] Gerencsér L., Maróti P. (2008). Eur. Biophys. J..

[bib11] Gerencsér L., Rinyu L., Kálmán L., Takahashi E., Wraight C.A., Maróti P. (2004). Acta Biol. Szeged..

[bib12] Giardi M.T., Koblízek M., Masojídek J. (2001). Biosens. Bioelectron..

[bib13] Giardi M.T., Pace E. (2005). Trends Biotechnol..

[bib14] Giustini M., Autullo M., Mennuni M., Palazzo G., Mallardi A. (2012). Sens. Actuators B.

[bib15] Goldsmith J.O., King B., Boxer S.G. (1996). Biochemistry.

[bib16] Hayes T.B., Khoury V., Narayan A., Nazir M., Park A., Brown T., Adam L., Chan E., Buchholz D., Stueve T., Gallipeau S. (2010). Proc. Natl. Acad. Sci. USA.

[bib17] Hayes T.B., Collins A., Lee M., Mendoza M., Noriega N., Stuart A.A., Vonk A. (2002). Proc. Natl. Acad. Sci. USA.

[bib18] Hayes T.B., Anderson L.L., Beasley V.R., de Solla S.R., Iguchi T., Ingraham H., Kestemont P., Kniewald J., Kniewald Z., Langlois V.S., Luque E.H., McCoy K.A., Muñoz-de-Toroj M., Oka T., Oliveira C.A., Orton F., Ruby S., Suzawa M., Tavera-Mendoza L.E., Trudeau V.L., Victor-Costa A.B., Willingham E. (2011). J. Steroid Biochem. Mol. Biol..

[bib19] Jablonowski N.D., Schäffer A., Burauel P. (2011). Environ. Sci. Pollut. Res..

[bib20] Jockers R., Bier F.F., Schmid R.D. (1993). Anal. Chim. Acta.

[bib21] Jones M.R. (2009). Biochem. Soc. Trans..

[bib22] Jones M.R., Fowler G.J.S., Gibson L.C.D., Grief G.G., Olsen J.D., Crielaard W., Hunter C.N. (1992). Mol. Microbiol..

[bib23] Jones M.R., Heer-Dawson M., Mattioli T.A., Hunter C.N., Robert B. (1994). FEBS Lett..

[bib24] Jones M.R., Visschers R.W., van Grondelle R., Hunter C.N. (1992). Biochemistry.

[bib25] Katona G., Snijder A., Gourdon P., Andréasson U., Hansson Ö., Andréasson L.-E., Neutze R. (2005). Nat. Struct. Mol. Biol..

[bib26] Koblížek M., Malý J., Masojídek J., Komenda J., Kučera T., Giardi M.T., Mattoo A.K., Pilloton R. (2002). Biotechnol. Bioeng..

[bib27] Köhler H.-R., Triebskorn R. (2013). Science.

[bib28] Kunze B., Kemmer T., Hofle G., Reichenbach H. (1984). J. Antibiot..

[bib29] Lambreva M.D., Giardi M.T., Rambaldi I., Antonacci A., Pastorelli S., Bertalan I., Husu I., Johanningmeier U., Rea G. (2013). PLoS ONE.

[bib30] Lancaster C.R.D., Michel H. (1997). Structure.

[bib31] Lancaster C.R.D., Michel H. (1999). J. Mol. Biol..

[bib32] Lancaster C.R., Bibikova M.V., Sabatino P., Oesterhelt D., Michel H. (2000). J. Biol. Chem..

[bib33] Mallardi A., Giustini M., Lopez F., Dezi M., Venturoli G., Palazzo G. (2007). J. Phys. Chem. B.

[bib34] Masojídek J., Souček P., Máchová J., Frolík J., Klem K., Malý J. (2011). Ecotoxicol. Environ. Saf..

[bib35] McAuley-Hecht K.E., Fyfe P.K., Ridge J.P., Prince S.M., Hunter C.N., Isaacs N.W., Cogdell R.J., Jones M.R. (1998). Biochemistry.

[bib36] McComb J.C., Stein R.R., Wraight C.A. (1990). Biochim. Biophys. Acta.

[bib37] Nakamura C., Hasegawa M., Nakamura N., Miyake J. (2003). Biosens. Bioelectron..

[bib38] Okamura M.Y., Steiner L.A., Feher G. (1974). Biochemistry.

[bib39] Paddock M.L., Rongey S.H., Abresch E.C., Feher G., Okamura M.Y. (1988). Photosynth. Res..

[bib40] Peters H., Schmidt-Dannert C., Schmid R.D. (1997). Mat. Sci. Eng. C.

[bib41] Rea G., Polticelli F., Antonacci A., Scognamiglio V., Katiyar P., Kulkarni S.A., Johanningmeier U., Giardi M.T. (2009). Protein Sci..

[bib42] SANCO/10496/2003, January 2014. 〈http://ec.europa.eu/food/plant/protection/evaluation/existactive/list_atrazine.pdf〉.

[bib43] Scognamiglio V., Pezzotti G., Pezzotti I., Cano J., Buonasera K., Giannini D., Giardi M.T. (2010). Microchim. Acta.

[bib44] Scognamiglio V., Stano P., Polticelli F., Antonacci A., Lambreva M.D., Pochetti G., Giardi M.T., Rea G. (2013). Phys. Chem. Chem. Phys..

[bib45] Sinning I., Michel H., Mathis P., Rutherford A.W. (1989). FEBS Lett..

[bib46] Spyridaki A., Fritzsch G., Kouimtzoglou E., Baciou L., Ghanotakis D. (2000). Biochim. Biophys. Acta.

[bib47] Stein R.R., Castellvi A.L., Bogacz J.P., Wraight C.A. (1984). J. Cell Biochem..

[bib48] Straley S.C., Parson W.W., Mauzerall D.C., Clayton R.K. (1973). Biochim. Biophys. Acta.

[bib49] Tibuzzi A., Rea G., Pezzotti G., Esposito D., Johanningmeier U., Giardi M.T. (2007). J. Phys. Condens. Matter.

[bib50] Touloupakis E., Giannoudi L., Piletsky S.A., Guzzella L., Pozzoni F., Giardi M.T. (2005). Biosens. Bioelectron..

[bib51] Victor-Costa A.B., Bandeira S.M.C., Oliveira A.G., Mahecha G.A.B., Oliveira C.A. (2010). Reprod. Toxicol..

