# Supplementary material for: Evaluation of a biohybrid photoelectrochemical cell employing the purple bacterial reaction centre as a biosensor for herbicides
Source: Biosens Bioelectron. 2014 Aug 15;58(100):172–8. doi: 10.1016/j.bios.2014.02.050 (PMC4009402; doi:10.1016/j.bios.2014.02.050)
Supplement: Supplementary file 1 — Supplementary material [file mmc1.docx]

SUPPLEMENTARY INFORMATION

**Evaluation of a biohybrid photoelectrochemical cell employing the purple bacterial reaction centre as a biosensor for herbicides.**

David J. K. Swainsbury^a^, Vincent M. Friebe^b^, Raoul N. Frese^b^ and Michael R. Jones^a^

*^a^ School of Biochemistry, Medical Sciences Building, University of Bristol, University Walk, Bristol BS8 1TD, United Kingdom*

*^b^ Division of Physics and Astronomy, Department of Biophysics, VU University Amsterdam, De Boelelaan 1081, Amsterdam 1081 HV, The Netherlands*

**
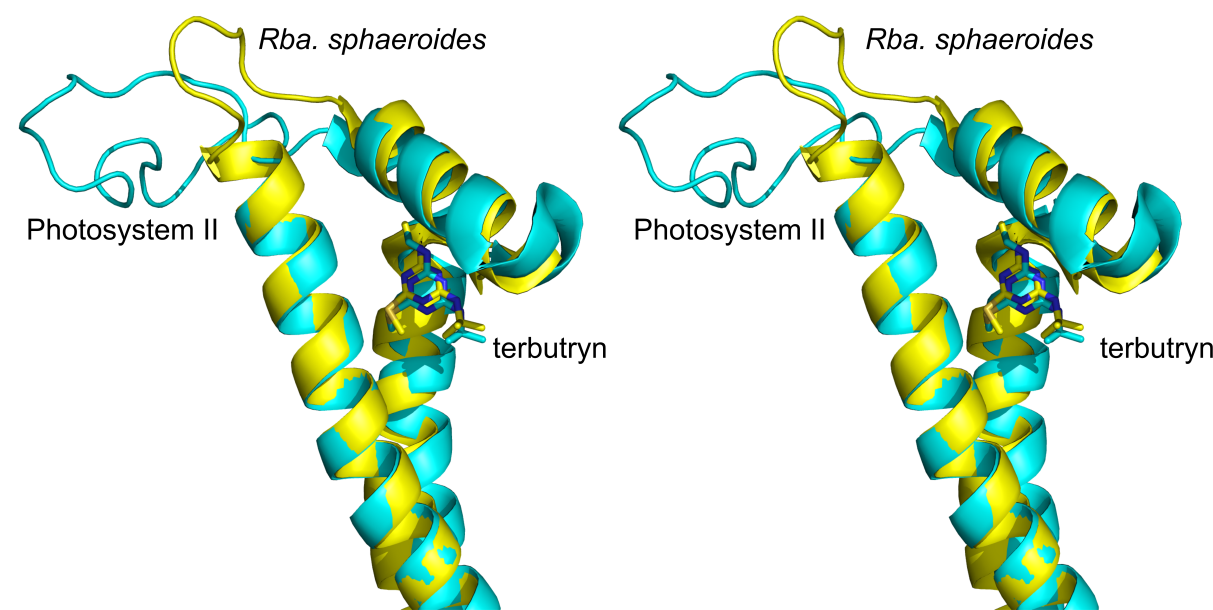
**

**Supplementary Fig. 1.** Stereo view of overlay of the D, E and de ɑ-helices that form the Q_B_ pocket, and terbutryn occupying the pocket in the *Rba. sphaeroides* reaction centre (yellow) and the *T. elongatus* PSII (cyan). Prepared using Protein Data Bank entries 2BNP (Katona et al. 2005) and 3PRQ (Broser et al. 2010), and PyMOL (Schrödinger, LLC).

**
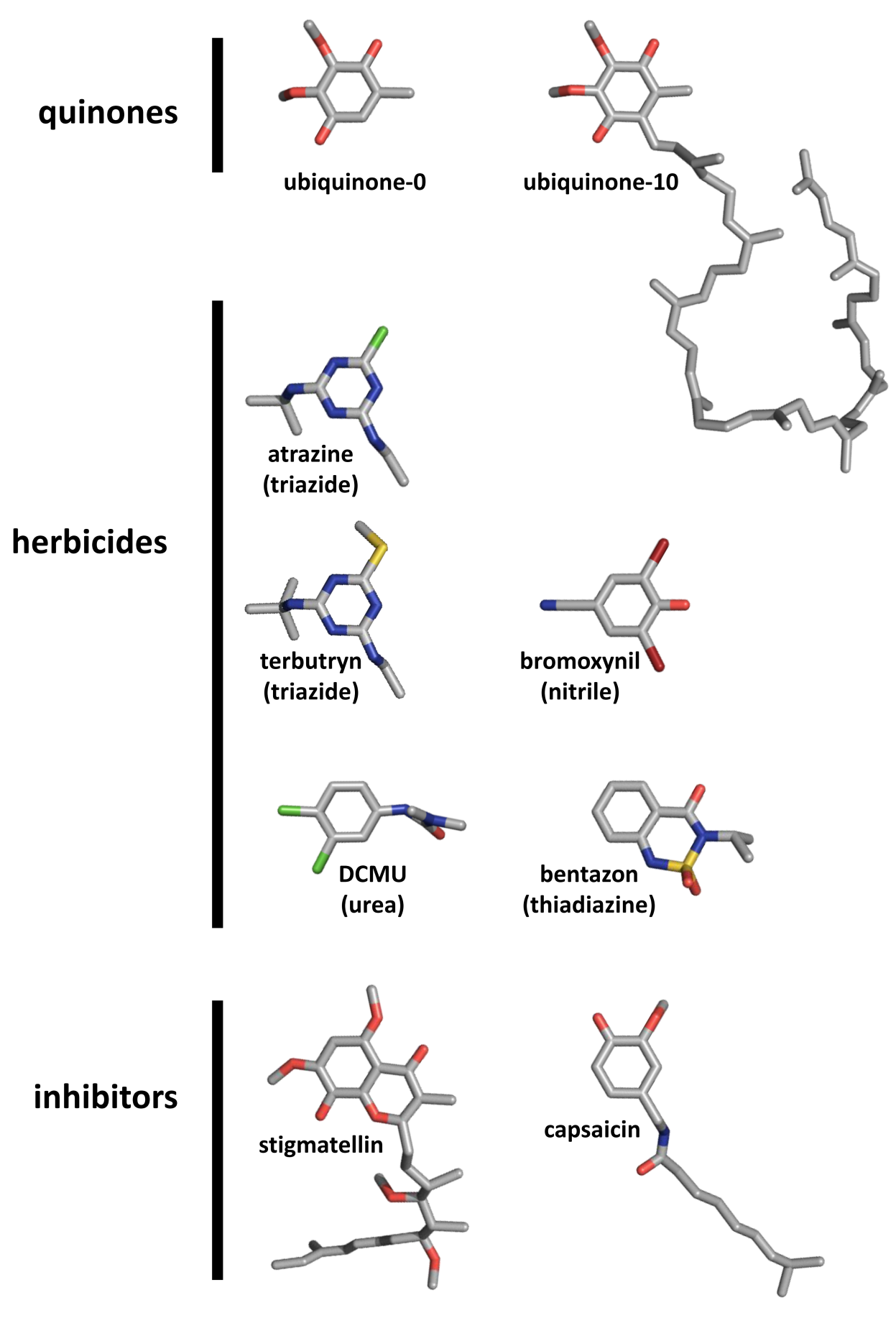
**

**Supplementary Fig. 2.** Structures of the quinones, herbicides and inhibitors used in this work. Three dimensional structures are from the ChemSpider database (Royal Society of Chemistry) and are for ubiquinone-0 (ChemSpider ID 62289), ubiquinone-10 (4445197), atrazine (2169), terbutryn (12874), bentazon (2238), bromoxynil (14775), DCMU (3008), stigmatellin (394850) and capsaicin (1265957). Atom colours are: carbon – grey, oxygen – red, nitrogen – blue, chloride – green, sulphur – yellow.


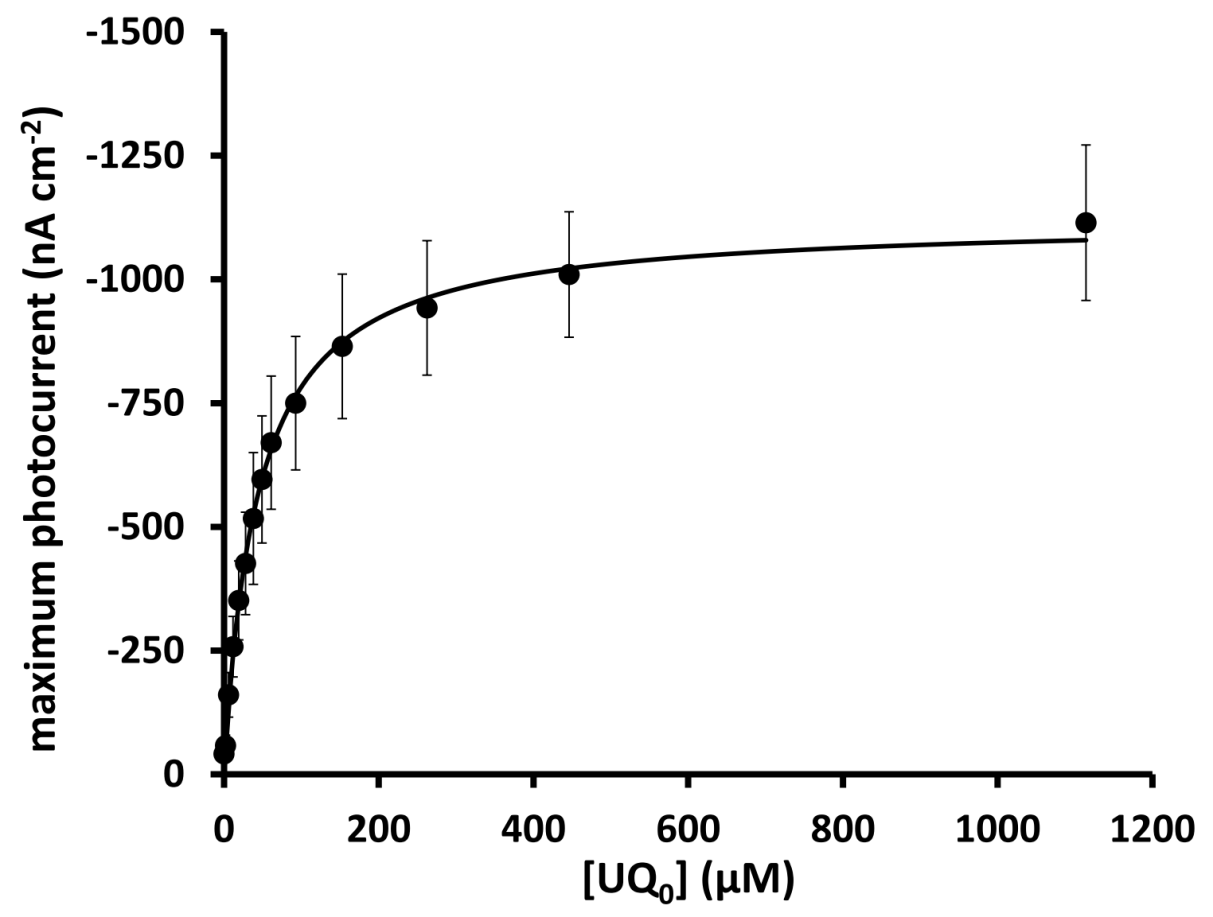


**Supplementary Fig. 3.** Maximum photocurrent densities as a function of UQ_0_ concentration. Points are an average of three data sets with standard errors. The solid line shows the fit to the Michaelis–Menten equation; *K_m_*^(apparent)^ = 42 ± 2 µM and maximum current density = -1106 ± 16 nA cm^-2^.

**Supplementary Table 1.** Effects of inhibitors on charge recombination.

| **Inhibitor**  (500 µM) | **τ_1_^a^**  (s) |  | **A_1_^a^**  (%) | **τ_2_**  (s) | **A_2_**  (%) |
| --- | --- | --- | --- | --- | --- |
|  |  |  |  |  |  |
| none | 0.096 ± 0.014 |  | 62.2 ± 3.1 | 0.96 ± 0.03 | 37.8 ± 7.3 |
| atrazine | 0.212 ± 0.004 |  | 100 | --- | --- |
| terbutryn | 0.152 ± 0.004 |  | 100 | --- | --- |
| stigmatellin | 0.095 ± 0.003 |  | 100 | --- | --- |
| bentazon | 0.129 ± 0.015 |  | 39.0 ± 5.8 | 1.09 ± 0.03 | 61.0 ± 3.0 |
| bromoxynil | 0.038 ± 0.009 |  | 36.1 ± 12.4 | 1.14 ± 0.04 | 63.9 ± 2.2 |
| capsaicin | 0.152 ± 0.011 |  | 41.0 ± 3.8 | 1.19 ± 0.03 | 59.0 ± 2.2 |
| DCMU | 0.179 ± 0.012 |  | 51.7 ± 3.7 | 1.18 ± 0.04 | 48.3 ± 3.7 |

All values are given ± standard error.

**^a^** Variations in τ_1_ and A_1_ should be treated with caution given the level of noise in the experimental data and the duration of the excitation pulse (~50 ms) relative to this time constant.
